# VDAC1 Knockout Affects Mitochondrial Oxygen Consumption Triggering a Rearrangement of ETC by Impacting on Complex I Activity

**DOI:** 10.3390/ijms24043687

**Published:** 2023-02-12

**Authors:** Andrea Magrì, Salvatore Antonio Maria Cubisino, Giuseppe Battiato, Cristiana Lucia Rita Lipari, Stefano Conti Nibali, Miriam Wissam Saab, Alessandra Pittalà, Angela Maria Amorini, Vito De Pinto, Angela Messina

**Affiliations:** 1 Department of Biological, Geological and Environmental Sciences, University of Catania, Via S. Sofia 64, 95125 Catania, Italy; 2 we.MitoBiotech S.R.L., C.so Italia 174, 95125 Catania, Italy; 3 Department of Biomedical and Biotechnological Sciences, University of Catania, Via S. Sofia 64, 95125 Catania, Italy; 4 Department of Biomedical and Biotechnological Sciences, Division of Medical Biochemistry, University of Catania, Via S. Sofia 97, 95123 Catania, Italy

**Keywords:** mitochondria, VDAC1 knockout, HAP1 cells, complex I, respiratory reserve(s)

## Abstract

Voltage-Dependent Anion-selective Channel isoform 1 (VDAC1) is the most abundant isoform of the outer mitochondrial membrane (OMM) porins and the principal gate for ions and metabolites to and from the organelle. VDAC1 is also involved in a number of additional functions, such as the regulation of apoptosis. Although the protein is not directly involved in mitochondrial respiration, its deletion in yeast triggers a complete rewiring of the whole cell metabolism, with the inactivation of the main mitochondrial functions. In this work, we analyzed in detail the impact of VDAC1 knockout on mitochondrial respiration in the near-haploid human cell line HAP1. Results indicate that, despite the presence of other VDAC isoforms in the cell, the inactivation of VDAC1 correlates with a dramatic impairment in oxygen consumption and a re-organization of the relative contributions of the electron transport chain (ETC) enzymes. Precisely, in VDAC1 knockout HAP1 cells, the complex I-linked respiration (N-pathway) is increased by drawing resources from respiratory reserves. Overall, the data reported here strengthen the key role of VDAC1 as a general regulator of mitochondrial metabolism.

## 1. Introduction

The Voltage-Dependent Anion-selective Channel (VDAC) is the most abundant and ubiquitously expressed family of pore-forming proteins of the outer mitochondrial membrane (OMM). Allowing for the passive diffusion of small, hydrophilic molecules up to 5000 Daltons, the VDAC proteins guarantee the permeability of the OMM by regulating the metabolic cross-talk between the mitochondria and the rest of the cell [1,2]. VDAC owes its name to its peculiar electrophysiological properties, established in artificial membrane reconstitution experiments more than three decades ago: the channel conductance changes according to the voltage applied, switching from high-conducting and anion-selective state to low-conducting and cation-selective states [3,4,5].

Three genes encode three highly conserved isoforms in mammals. They are named VDAC1, VDAC2, and VDAC3 in agreement with the order of their discovery [6]. The tridimensional structures of mouse or human VDAC1 [7,8,9] and zebrafish VDAC2 [10] are available, and share a common β-barrel transmembrane pore, made of 19 antiparallel β-strands, and an N-terminal domain organized as α-helix, confirmed also by in vitro experiments [11]. Among the isoforms, VDAC1 is the most abundant, overcoming VDAC2 and VDAC3 by one or two orders of magnitude, respectively [12].

Being at the interface between cytosol and mitochondria, VDAC1 mainly regulates the flux of Krebs’ cycle intermediates (pyruvate, succinate, malate, and glutamate), ADP/ATP, ions (Na^+^, K^+^, Cl^−^), nucleotides, and NAD^+^/NADH, thus supporting the whole cellular bioenergetics [13,14,15,16]. Furthermore, VDAC1 acts as an anchor for many cytosolic proteins, including Hexokinases and specific members of the Bcl-2 family, participating in the regulation of mitochondrial-mediated apoptosis [17,18,19,20]. In light of these considerations, VDAC1 has rapidly become a pharmacological target in cancer and neurodegeneration [21,22,23,24].

Similar to isoform 1, VDAC2 is a key modulator of cell death and survival, exerting both pro- and anti-apoptotic functions by interacting with Bak and Bax [25,26,27]. The role of VDAC3 appears to be more intricate: it has specific electrophysiological features and post-translational modifications that significantly change with the redox environment [28,29,30]. Only recently, however, has our group definitely demonstrated that VDAC3 is a redox-sensing protein, protecting mitochondria from oxidative stress [31].

Much information about VDAC functions derives from gene inactivation studies. In the yeast *S. cerevisiae*, endowed with a single functional mitochondrial porin [32], inactivation of VDAC1 dramatically affects mitochondrial DNA maintenance and expression and organelle functioning, leading to a complete rewiring of the whole cell metabolism [33]. In mammals, knockout experiments are made more complicated to interpret due to the presence of the other isoforms, which can partially complement the lack of VDAC1. Interestingly, in mouse embryonic fibroblast VDAC1 deletion promotes a partial rearrangement of gene expression that triggers metabolic impairments and the accumulation of reactive oxygen species (ROS) [34], overlapping in part the changes already seen in yeast. Additionally, VDAC1 knockout makes H9c2 cells more susceptible to ROS-induced apoptosis [35], while in chronic myelogenous leukemia-derived cells it correlates with a slight reduction in mitochondrial mass and the hyperpolarization of the mitochondrial membranes [31]. Nevertheless, despite the important role played by VDAC1 for mitochondrial metabolism, its possible involvement in the regulation of mitochondrial respiration has not been adequately investigated yet.

Aimed at studying the above-mentioned mechanism, the near-haploid human HAP1 cells knockout for VDAC1 were used to conduct high-resolution respiration (HRR) experiments. Results clearly indicated that the lack of VDAC1 or its partial inhibition correlates with a reduction of oxygen consumption in the main respiratory states. At the same time, VDAC1 knockout cells undergo a re-arrangement of the respiration by increasing the relative efficiency of complex I-linked respiration, dissipating part of respiratory reserves. Overall, these findings remark on the pivotal role of VDAC1 as a key regulator of mitochondrial functionality.

## 2. Results

### 2.1. Characterization of HAP1 ΔVDAC1 Cells

**Figure 1:**
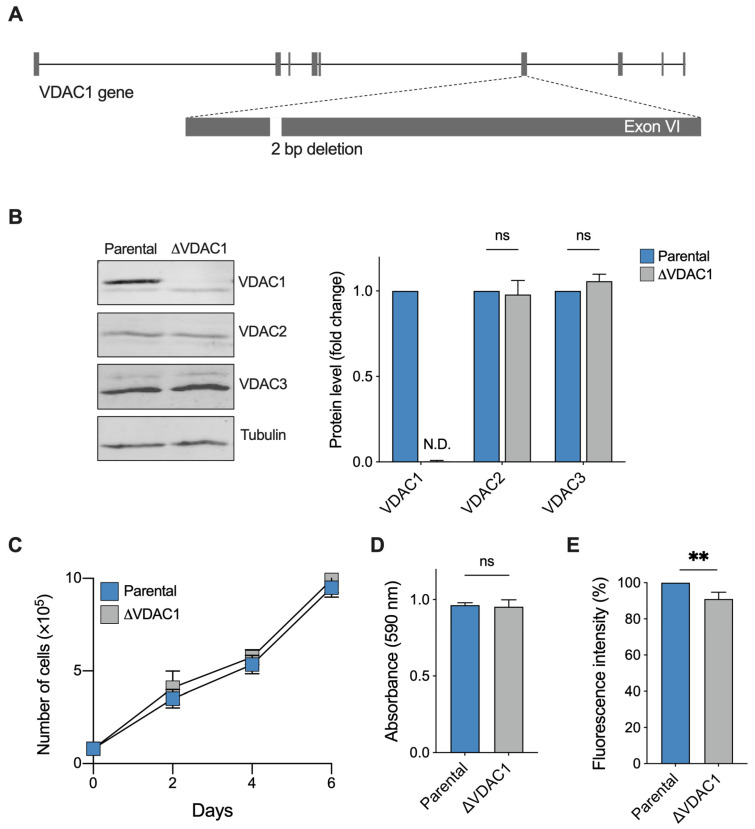
Phenotypical characterization of HAP1 cells used in this work. (**A**) Schematization of VDAC1 knock-out: a 2 bp deletion in the exon VI of VDAC1 gene was performed. (**B**) Representative Western blotting (left) and the relative protein level quantification (right) of total lysates from parental and ΔVDAC1 cells, showing the expression of the three VDAC isoforms. As expected, no VDAC1 protein band was detected in knock-out cells. N.D.—not detectable. (**C**) Proliferation assay showing no significant difference between samples. Data are shown as mean ± SD of *n* = 3 independent counts and statistically analyzed by two-way ANOVA; ns—not significant. (**D**) Cell viability analysis by MTT assay showing no significant difference between samples. (**E**) Quantification of the MitoTraker signal in parental and ΔVDAC1 cells by flow cytometry. A slight but significant difference of approximately 10% was observed. Data in (**D**,**E**) are expressed as mean ± SEM of *n* = 3 independent counts and statistically analyzed by unpaired *t*-test, with ** *p* < 0.01; ns—not significant.

HAP1 cells are endowed with a single copy of each chromosome and represent a powerful model for knockout studies [36]. As schematized in Figure 1A, the inactivation of VDAC1 expression was achieved by a deletion of 2 bp in exon VI of the corresponding gene. In the resulting cell line (namely ΔVDAC1), no protein band was detected by anti-VDAC1 antibody in Western blot experiments (Figure 1B). In contrast, cells of the parental line, expressing the wild type VDAC1 protein (here used as reference control), showed an easily detectable band at the expected molecular weight of ~32 kDa (Figure 1B). In addition, the expression level of the VDAC2 and VDAC3 isoforms was also tested. In this regard, no significant differences were detected between ΔVDAC1 and the parental cells, as confirmed by quantification (Figure 1B). Thus, unlike in other cell lines [12], the non-expression of VDAC1 in HAP1 cells does not affect the expression of the other VDAC isoforms. Similarly to what was previously observed [31], VDAC1 knockout did not alter cell proliferation or viability: as shown in Figure 1C, the growth curve of the ΔVDAC1 HAP1 cells is almost overlapped to the parental one; furthermore, no significant differences were observed upon MTT assay (Figure 1D). Contrariwise, a slight but significant reduction in the mitochondrial mass was confirmed in knockout cells of −10.5%, in comparison to parental HAP1 (*p* = 0.0012, *n* = 3), as revealed by the quantification of MitoTraker fluorescence signal by flow cytometry (Figure 1E).

### 2.2. Deletion of VDAC1 Gene or Inhibition of Its Protein Affects Oxygen Consumption of HAP1 Cells

**Figure 2:**
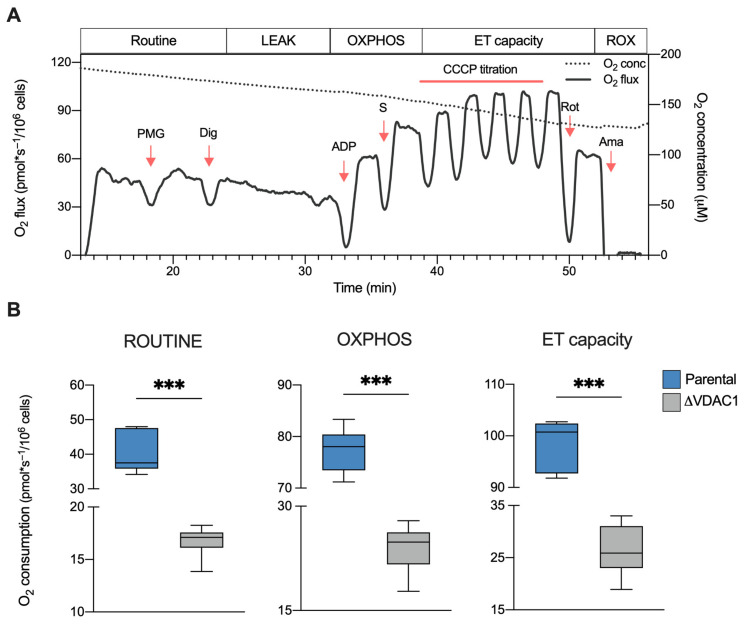
Oxygen consumption in HAP1 cells. (**A**) Representative curve of mitochondrial respiratory profile of parental HAP1 cells along with the SUIT protocol used for both intact and permeabilized cells. P—pyruvate; M—malate; G—glutamate; Dig—digitonin; S—succinate; Rot—rotenone; Ama—antimycin. (**B**) Quantitative analysis of the oxygen consumption rate of ROUTINE, OXPHOS, and maximal ET capacity in HAP1 cells, expressed as pmol/second per million cells. A significant reduction of oxygen consumption was observed in each state for ΔVDAC1 cells in comparison to parental. Data are shown as median ± SEM of *n* = 6 independent experiments and statistically analyzed by unpaired *t*-test, with *** *p* < 0.001.

The overall oxygen consumption of HAP1 cells was investigated by HRR. This technique allows for the measurement of oxygen flow relative to different respiratory states, as well as the contribution of each state or specific electron transport chain (ETC) complexes to the achievement of the maximal respiratory capacity [37]. For this purpose, a Substrate-Uncoupler-Inhibitor-Titration (SUIT) protocol was used as schematized in Figure 2A in addition to a representative curve of HAP1 parental cells. Particularly, the curve shows variations in the oxygen consumption upon the addition of specific molecules. Briefly, respiration in the presence of endogenous substrates (ROUTINE state) was measured in intact cells; then, through a mild permeabilization of plasma membranes and stimulation with specific substrates and ADP, the oxidative phosphorylation-linked respiration (OXPHOS state) was assessed; finally, the maximal electron input to electron transport (ET) chain was determined by titration with an uncoupler.

The same protocol was then applied to ΔVDAC1 cells, and the oxygen consumption in the main respiratory states was calculated and compared with the control (see Supplementary Figure S1 for a representative curve of ΔVDAC1 cells). As displayed in Figure 2B, the knockout of VDAC1 dramatically affects the oxygen flows in all the analyzed states. Precisely, ΔVDAC1 cells displayed about 58% of flux reduction in intact cells (ROUTINE) and of about 68% after cell permeabilization and the stimulation of the OXPHOS activity (*p* < 0.001 vs. parental HAP1, *n* = 6). Additionally, the oxygen consumption linked to the maximal capacity was reduced by approximately 76% (*p* < 0.001 vs. parental HAP1, *n* = 6).

**Figure 3:**
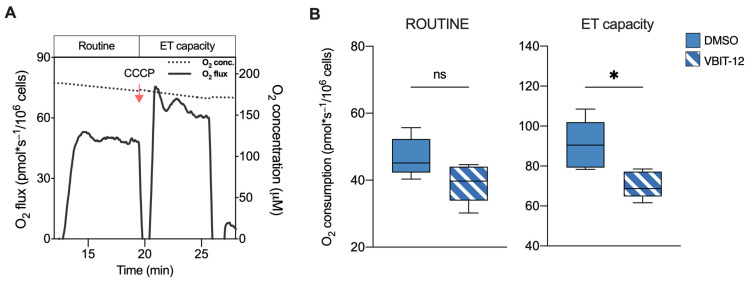
Oxygen consumption in HAP1 cells upon exposure to VBIT-12. (**A**) A representative curve of mitochondrial respiratory profile of parental HAP1 intact cells along with the SUIT protocol here used. Uncoupler CCCP was added for the achievement of maximal respiratory capacity. (**B**) Quantitative analysis of the oxygen consumption rate of ROUTINE and maximal ET capacity in HAP1 parental cells previously treated with VBIT-12 or DMSO (control). A significant reduction of ET capacity was observed in treated cells. Data are expressed as pmol/second per million cells and shown as median ± SEM of *n* = 6 independent experiments. Data were statistically analyzed by unpaired *t*-test, with * *p* < 0.05; ns—not significant.

On the basis of these results, we queried whether these reductions were directly due to the VDAC1 inactivation or to VDAC1-independent changes occurring in our knockout model. To this aim, parental HAP1 cells were exposed to a sublethal dose of VBIT-12, a specific VDAC1 inhibitor. VBIT are a group of small, cell-penetrating molecules able to interact with VDAC1 that are known for their ability to reduce the channel conductance [38,39]. In this specific case, an HRR analysis was limited to intact cells by measuring the ROUTINE state and maximal ET capacity (Figure 3A). Notably, these parameters were evaluated after a rapid treatment with VBIT-12 to avoid any eventual side effects due to the prolonged exposure to the molecule. As reported in Figure 3B, a significant reduction of the respiration was observed in VBIT-12 treated cells, consisting of −22% for the ET capacity (*p* = 0.01 vs. DMSO treated cells, *n* = 6). Although not significant, a similar decreasing trend was observed also for ROUTINE state (*p* = 0.07, *n* = 6). On the contrary, no significant effect of VBIT-12 was noticed in the ΔVDAC1 cells exposed to the molecule (Supplementary Figure S2), suggesting that VBIT-12 exerts its inhibitory action on VDAC1 specifically.

Taken together, these results clearly support a pivotal role of VDAC1 in the regulation of mitochondrial respiration.

### 2.3. VDAC1 Knockout Changes the Contribution of Respiratory States or Complexes to ET Capacity

**Figure 4:**
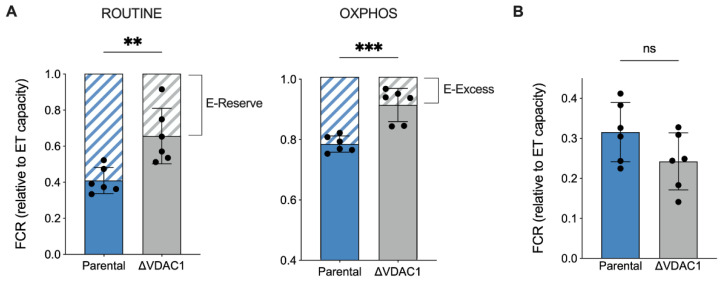
Analysis of flux control ratios (FCRs) in HAP1 cells. (**A**) The specific contribution of ROUTINE and OXPHOS states to ET capacity was calculated and demonstrated as FCRs, along with the respiratory reserves (dashed histograms), E-Reserve, and E-Excess. A significant increase in both FCR values was observed for ΔVDAC1 cells, which correspond to a proportional decrement of the reserves. (**B**) FCRs calculated for LEAK state. No significant differences were detected between parental and ΔVDAC1 cells. Data in (**A**,**B**) are shown as mean ± SEM of *n* = 6 independent experiments and statistically analyzed by unpaired *t*-test, with ** *p* < 0.01 and *** *p* < 0.001; ns—not significant.

Next, the relative contribution of each respiratory state or ETC complex to the achievement of the maximal capacity was investigated in an independent manner from mitochondrial mass or other external factors by analyzing the flux control ratios (FCRs) [37,40]. In Figure 4A, the relative contributions of ROUTINE and OXPHOS to the maximal respiration are shown. Surprisingly, the ROUTINE contribution was higher in ΔVDAC1 cells (+37%, *p* = 0.0053 vs. parental HAP1, *n* = 6). In a similar manner, the relative contribution of OXPHOS was increased upon VDAC1 knockout of about 15% (*p* = 0.004 vs. parental HAP1, *n* = 6). Notably, the approach of ROUTINE and OXPHOS to the maximal respiration correlates with a significant reduction of the respiratory reserve (E-Reserve) and excess (E-Excess), respectively (Figure 4A, dashed histograms), that consist of bioenergetic supplies to which mitochondria can draw in the presence of further stimuli to produce extra ATP [41].

Based on previous results, we queried whether increases in the ROUTINE and OXPHOS contributions were due to a proportional raise in the non-phosphorylating component, the so-called LEAK state, here measured after permeabilization of the plasma membranes, i.e., allowing ADP to leave the cells. As reported in Figure 4B, however, the LEAK contribution was unvaried among the samples.

**Figure 5:**
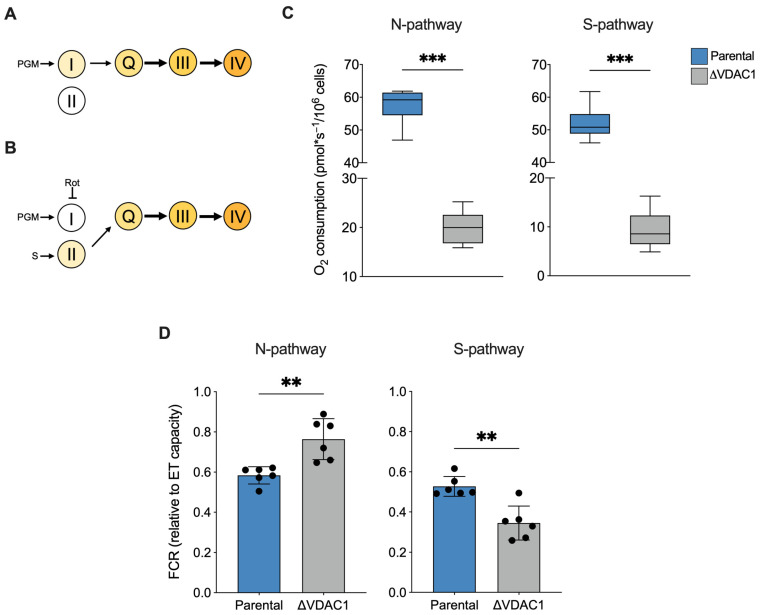
Analysis of N- and S-pathways in HAP1 cells. (**A**,**B**) Schematization of ET chain activity in our experimental HRR setup. Activation of complex III via Q-junction can occur exclusively by complex I or II, precisely by stimulating complex I with NADH-linked substrates and in the absence of succinate (N-pathway, **A**) or by stimulating complex II with succinate upon inhibition of complex I with rotenone (S-pathway, **B**). P—pyruvate; M—malate; G—glutamate; S—succinate; Rot—rotenone. (**C**) Quantitative analysis of the oxygen consumption rate of N- and S-pathway expressed as pmol/second per million cells. A significant reduction of oxygen consumption was observed in each state for ΔVDAC1 cells in comparison to parental. (**D**) FCRs calculated for N- and S-pathways revealed an increasement of N- over the S-pathway in ΔVDAC1 cells. Data are shown as median (**C**) or means (**D**) ±SEM of *n* = 6 independent experiments and statistically analyzed by unpaired *t*-test, ** *p* < 0.01 and *** *p* < 0.001.

Next, we analyzed the respiration linked to the NADH-substrates (N-pathway) or succinate (S-pathway), namely the respiration coupled to complex I or II, respectively. Precisely, the N-pathway was achieved in the presence of saturating concentrations of pyruvate, malate, glutamate, and ADP, but not succinate. As schematized in Figure 5A, in this configuration electrons flow exclusively from complex I to complex III through the Q-junction. Alternatively, electrons may flow from complex II to the Q-junction in the presence of a saturating concentration of succinate and rotenone, a specific inhibitor of complex I (Figure 5B).

In term of oxygen consumption, the both N- and S-pathways followed a reduction in line with the typical respiratory profile of ΔVDAC1 cells (−65% and −82% respectively, *p* < 0.001 vs. parental HAP1, *n* = 6, Figure 5C). In addition, interesting differences emerged from a FCR analysis. Particularly, as displayed in Figure 5D, a significant increase in the relative contribution of the N-pathway was observed in the ΔVDAC1 cells (+24%, *p* = 0.0026 vs. parental HAP1, *n* = 6), counterbalanced by a reduction in the relative contribution of the S-pathway (−34%, *p* < 0.001 vs. parental HAP1, *n* = 6).

Overall, the FCR analysis suggests that the increase in contribution of the ROUTINE and OXPHOS respiration previously observed in ΔVDAC1 cells is strictly dependent from complex I activity.

### 2.4. Mitochondrial Oxidation of NADH Is Increased in VDAC1 Knockout Cells

**Figure 6:**
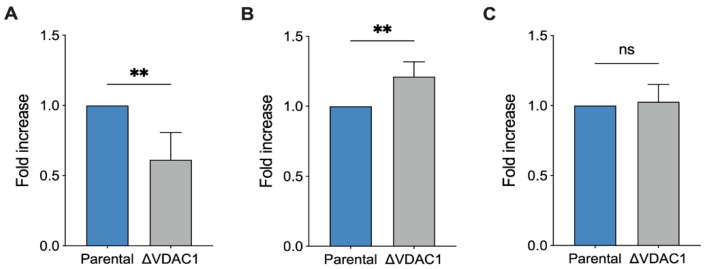
Analysis of NADH reduction in HAP1 cells. Quantitative analysis of total level of nicotinamide cofactors NAD^+^, NADH, NADP^+^, and NADPH (**A**), the ratio NAD^+^/NADH (**B**) and the overall level of lactate (**C**). The lower level of nicotinamide cofactors in ΔVDAC1 cells is counterbalanced by high ratio of NAD^+^/NADH value. Data are expressed as fold increase using parental HAP1 as control. Data are shown as median ± SEM of *n* = 4 independent experiments and statistically analyzed by unpaired *t*-test, with ** *p* < 0.01; ns—not significant.

The cellular content of nicotinic coenzymes, in the form of phosphorylated and non-phosphorylated nicotinamide dinucleotides, renders a clear picture of both the redox status and the mitochondrial functionality. In this view, we analyzed the total concentration of the four different forms (NAD^+^, NADH, NADP^+^, and NADPH; see Table S1 for raw data) in extracts from parental and ΔVDAC1 HAP1 cells. As reported in Figure 6A, a dramatic decrease in the total nicotinic coenzyme pool was observed in the ΔVDAC1 sample (−40%, *p* = 0.007 vs. parental HAP1, *n* = 4).

Notably, this finding is in line with previous respirometric results, in which a modulation of mitochondrial efficiency was observed and, in particular, a new condition of limited oxygen utilization. Interestingly, the NAD^+^/NADH increased by 20% (*p* = 0.007 vs. parental HAP1, *n* = 4, Figure 6B), possibly suggesting a higher rate of NADH oxidation at the level of complex I, ensuring a proper function of either OXPHOS or ET capacity and, preliminarily of the TCA cycle. According to these data, we noticed no significant differences between our samples in lactate concentration (Figure 6C), suggesting that the increased oxidation of NADH in ΔVDAC1 cells is prevalently mitochondrial rather than cytosolic via lactate dehydrogenase [42].

### 2.5. VDAC1 Knockout Makes Cells More Sensitive to Rotenone but Not to Malonic Acid

In light of the previous observations, we finally assayed the effect of specific ETC complex inhibitors. For this purpose, a SUIT protocol developed for intact cells was used. Briefly, the maximal ET capacity of each sample was reached by CCCP; then, variations in the respiration were monitored by titration with inhibitors.

**Figure 7:**
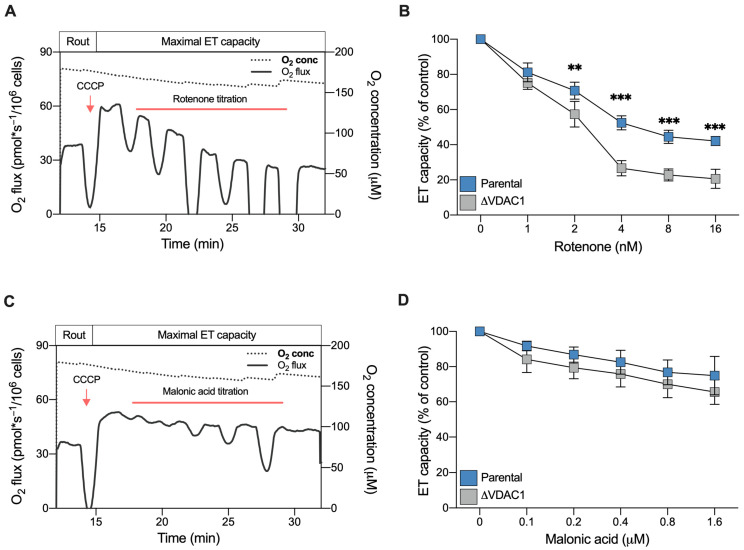
Sensitivity of HAP1 cells to complex I and II inhibitors assayed by HRR. (**A**) A representative curve of mitochondrial respiratory profile of parental HAP1 intact cells, in addition to the SUIT protocol used here. After achieving the of ET capacity with CCCP, respiration was monitored upon rotenone titration (1–16 nM). (**B**) Quantitative analysis of ET capacity upon rotenone titration showing a reduction for both parental and ΔVDAC1 cells. However, an increased sensitivity to rotenone was observed for ΔVDAC1 cells in comparison to the parental cells. (**C**) A representative curve of the mitochondrial respiratory profile of parental HAP1 intact cells, in addition to the SUIT protocol used here for malonic acid titration (0.1–1.6 μM). (**D**) Quantitative analysis showing reduction of ET capacity upon titration with malonic acid. No significant difference in the respirometric profile was observed for parental and ΔVDAC1 cells. Data in (**B**,**D**) are shown as means ± SEM of *n* = 3 independent experiments and statistically analyzed by a two-way ANOVA, with ** *p* < 0.01 and *** *p* < 0.01.

Rotenone is a lipophilic, cell-permeable molecule, commonly used as a specific inhibitor of complex I and as toxin for mimicking molecular features of Parkinson’s disease [43]. Figure 7A shows a representative curve of HAP1 parental cells along with the SUIT protocol applied here. As reported, titration with non-saturating doses of rotenone in the nanomolar order induced a progressive reduction of the ET capacity in a dose–response manner, as expected. Particularly, at higher concentrations used here (16 nM), the ET capacity was reduced by approximately 50% in comparison to the untreated control (Figure 7B). A similar dose–response effect was observed with the ΔVDAC1 cells. However, in this specific case, the titration effect was more pronounced: not only was 2 nM of rotenone sufficient to halve ET capacity (*p* = 0.0046 vs. parental HAP1, *n* = 3) but, at higher concentrations, the toxin reduced the ET capacity by up to 80% of its original value (*p* < 0.001 vs. parental HAP1, *n* = 3, Figure 7B).

We repeated the experiment using malonic acid, which specifically inhibits complex II. As reported in Figure 7C,D, titration with malonic acid was better tolerated by HAP1 cells, both parental and VDAC1 knockout. In fact, at the highest concentration used here, the maximal inhibition achieved was about 30% in both samples. Regardless, no significant differences were observed between the parental and knockout cells (Figure 7D).

Once again, these results indicate the importance of complex I contribution to respiration for ΔVDAC1 cells bioenergetic.

## 3. Discussion

Mitochondrial VDACs allow for the passive diffusion of the majority of substrates, feeding the Krebs’s cycle and the respiratory enzymes of the ETC [1,17]. Moreover, they represent the most abundant proteins in the OMM, conferring its typical, sieve-like aspect, as revealed by atomic force microscopy experiments [44,45]. In the yeast *S. cerevisiae*, where the complete mitochondrial proteome was characterized, up to 19.000 copies of VDAC1 per single mitochondrion were estimated, an amount that exceeds by approximately ten times the copy number of the second most abundant protein, Tom40 [46]. For these reasons, VDACs are widely considered key players in the maintenance of the metabolic exchanges across the OMM.

Interested in characterizing the precise role of the main isoform, VDAC1, in respiration, a specific function of the mitochondrion, we used a VDAC1 knockout HAP1 cell line. The haploid line, HAP1, is a particularly advantageous model as gene knockout guarantees the total absence of the corresponding gene product. Specifically, the HAP1 ΔVDAC1 cell line we used is particularly suitable for our purposes because inactivation of the single copy of the VDAC1 gene does not lead to compensatory responses and subsequent changes in the expression levels of the other VDAC isoforms. In addition, this cellular model can be easily used in HRR experiments in both intact or permeabilized protocols, as recently demonstrated by our group and an independent group [31,47].

Many interesting outcomes emerged from the detailed HRR analysis performed here. Firstly, despite the presence of other two isoforms, VDAC1 appears to be the preferential route for metabolites to move in and out of the mitochondria. In fact, VDAC1 inactivation dramatically affects the oxygen consumption of HAP1 cells in all three main respiratory states. This result cannot be explained only by the slight reduction in the mitochondrial mass observed here and elsewhere [31], and is confirmed by results achieved in experiments with VBIT-12.

Secondly, the relative contributions of ROUTINE and OXPHOS respiration to the maximal capacity are significantly higher in ΔVDAC1 than in parental cells. Surprisingly, this occurs without a proportional increase in the non-phosphorylating component of respiration. Even under physiological conditions, the mitochondrial coupling efficiency is always below 100% due to the proton gradient fraction, which is generated by the ETC and dissipates during a process known as proton leak [48]. An increase in the LEAK state is a common consequence of mitochondrial malfunctioning, as observed in the case of inner mitochondrial membrane damages or the overexpression of Uncoupling proteins, the latter being used by the cell as a protective strategy in the event of ROS accumulation or other stress stimuli [49,50]. Not coincidentally, many pathological conditions correlate with an increased LEAK respiration, which is widely recognized as a hallmark of mitochondrial dysfunction [40,51,52,53,54]. However, this was not the case for the ΔVDAC1 cells, as the specific contribution of the LEAK state in both HAP1 lines was comparable.

Thirdly, in ΔVDAC1 cells, the N-pathway is reduced in a similar manner to the S-pathway or total OXPHOS in terms of absolute values. Contrariwise, its increased FCR value, and specifically the sustained ratio of NAD^+^/NADH in favor of the oxidized form, indicate a major contribution of complex I over complex II in activating the ETC in our setup. These data suggest that VDAC1 deficiency prompts the cells toward a re-arrangement of the mitochondrial metabolism which involves complex I. It is, indeed, no coincidence that ΔVDAC1 cells are more sensitive to rotenone but not to malonic acid, as demonstrated by our titration experiments.

Overall, this appears to be a strategy put in place by VDAC1 knockout cells to keep mitochondria still active, even when a pivotal protein such as VDAC1 is missing. Regardless, the process comes with a price: the knockout cells draw resources from the respiratory reserves that the mitochondria can rely on for the production of extra ATP during high-energy demands or in the presence of additional stress stimuli [41,55]. Similar to the increase in LEAK respiration, the limitation of respiratory reserves is emerging as a dysfunctional parameter and a common feature of many pathological conditions, precisely the neurodegenerative processes [51,56].

Comparable results were previously attained in the ΔPOR1 yeast cells, which are devoid of endogenous VDAC1. Two genes in the yeast encode two porin isoforms, both showing the typical VDAC pores features in artificial membranes [57,58]. However, only VDAC1 is constitutively expressed. In fact, the expression of isoform 2 is negligible and occurs mainly in the presence of external stimuli [46,59]. In this context, the entire metabolism is rewired: the expression of mitochondrial genes is completely inhibited as a consequence of the dramatic reduction of mitochondrial DNA copies, and the cell is forced to switch toward a lipid-based metabolism [33]. Of course, in HAP1 cells, the consequences of VDAC1 inactivation are less severe due to the presence of the other isoforms: if, in ΔPOR1, no alternatives to VDAC1 exist, higher eukaryotes VDAC2 and VDAC3 are concomitantly and constitutively expressed. They are finely regulated [60,61,62] and can partially compensate for the absence of VDAC1, as demonstrated by complementation assays [28,63]. This allows for not only metabolic exchanges, even limited, but also mitochondrial DNA maintenance and expression thanks to the import of nucleotides [31].

Interestingly, our results strengthen previous data regarding the existence of a functional link between VDAC proteins and specific enzymes located in the inner mitochondrial membrane (IMM), precisely, the adenine nucleotide carrier and the dicarboxylate carrier [64]. These observations emerged from the analysis of respiratory State 3 in isolated rat liver mitochondria and mitoplasts, i.e., mitochondria devoid of OMM, and thus of all VDAC isoforms. In mitoplasts, indeed, the oxygen consumption achieved in the presence of succinate was significantly lower than in the whole mitochondria, as was the sensitivity to specific inhibitors targeted to these IMM enzymes. Conversely, the addition of a VDAC-enriched OMM preparation to mitoplasts increased respiration and partially restored sensitivity to inhibitors [64]. Although our experimental conditions were different, in the presence of succinate and rotenone (S-pathway), we noticed a similar reduction of oxygen consumption. This suggests that complex II might also be affected by the lack of mitochondrial porin.

In conclusion, our work definitely indicates the key role of VDAC1 in respiration and remarks, once again, on the pivotal role of the main porin isoform in preserving the proper functioning of mitochondria.

## 4. Materials and Methods

### 4.1. Cell Lines Maintenance, Proliferation and Treatment

HAP1 are near-haploid human cells of leukemic origin. The HAP1 parental and knockout VDAC1 cell lines were purchased from Horizon Discovery (Waterbeach, UK). Cells were maintained in Iscove’s modified Dulbecco’s media (IMDM, GIBCO, Waltham, MA, USA), supplemented with 10% of fetal bovine serum (GIBCO) and penicillin/streptomycin antibiotic (GIBCO) in a controlled environment (37 °C and 5% CO_2_). The proliferation of the HAP1 parental and knockout cells was monitored over time (6 days, each 24 h) by microscopy using a Burker’s chamber. At day 0, 80.000 cells per genotype were seeded in 12-well plates. Three independent cell-counting assays were conducted.

For the inhibition of VDAC1 activity, HAP1 parental or VDAC1 knock-out cells were exposed to 20 μM of VBIT-12 previously dissolved in DMSO, or to DMSO as a control, for 30 min in a controlled environment (37 °C and 5% CO_2_) prior to HRR experiments.

### 4.2. Western Blotting Analysis

The total lysates from the parental and VDAC1 knock-out HAP1 cells were obtained from 10^6^ cells in a lysis buffer (50 mM Tris-HCl pH 7.4, 150 mM NaCl, EDTA 1 mM, 1% TRITON X-100, protease inhibitors cocktail). Protein samples were separated using SDS-PAGE electrophoresis and transferred to a PDVF membrane (GE Healthcare, Chicago, IL, USA). The membranes were blocked in 5% BSA in PBS with 0.1% Tween-20 at room temperature for 1h and incubated overnight at 4 °C with the following primary antibodies: VDAC1 (1:1000, Abcam, Cambridge, UK), VDAC2 (1:200, Abcam), VDAC3 (1:100, Abcam), and β-Tubulin (1:2000, Cell Signaling, Danvers, MA, USA). After washing, membranes were incubated with IRDye-conjugated secondary antibodies (1:25,000, Li-Cor Biosciences, Lincoln, NE, USA). Signals were detected by the Odyssey CLx Imaging System (Li-Cor Biosciences) and analyzed using Image Studio Lite software (Li-Cor Biosciences). The same software was used for quantification.

### 4.3. Cell Viability Assay

The viability of the parental and VDAC1 knock-out HAP1 cells was investigating using a 3-(4,5-dimethylthiazolyl-2)-2,5-diphenyltetrazoliumbromide (MTT) assay. Cells were plated in 96-well plates (10.000 cell/well), and MTT was added after 24 h to achieve a final concentration of 5 mg/mL. After incubation (37 °C, 3 h) and medium removal, the formazan crystals produced were dissolved in 100 μL of dimethyl sulfoxide. Absorbance at 590 nm was then determined using the Varioskan microplate reader (Thermo Fisher, Waltham, MA, USA). Three independent experiments were performed in triplicate.

### 4.4. Analysis of Mitochondrial Mass

The mitochondrial mass of the HAP1 lines was estimated by flow cytometry using a fluorescent probe whose accumulation into mitochondria is independent of the mitochondrial membrane potential. Adherent cells were incubated for 30 min at 37 °C with Krebs Ringer Buffered Saline (130 mM NaCl, 3.6 mM KCl, 10 mM HEPES, 2 mM NaHCO_3_, 0.5 mM NaH_2_PO_4_, 0.5 mM MgCl_2_, 1.5 mM CaCl_2_, 4.5 g/L glucose, pH 7.42) and supplemented with 300 nM of Mito tracker Green (Thermo Fisher). Cells were then collected and analyzed at 490/516 nm using the CyFlow ML flow cytometer (Partec) system [31,53]. Data were acquired and gated using the FCS Express 4 software (DeNovo). Three sets of independent experiments, each performed in triplicate, were performed by analyzing approximately 20.000 cells for each condition.

### 4.5. High-Resolution Respirometry (HRR)

The characterization of mitochondrial respiration in the HAP1 lines was performed by HRR in the two-chamber system O2k-FluoRespirometer (Oroboros Instruments, Innsbruck, Austria). Specific SUIT protocols, aimed at analyzing the principal respiratory states and/or the contribution of ET complexes to the maximal respiratory capacity, were adapted from [51,53,65].

The complete respiratory profile was attained as follows. The ROUTINE state was measured in intact cells in the presence of endogenous substrates. Then, cells were subjected to a mild permeabilization of the plasma membranes with 3 μM digitonin. The dissipative respiration, the LEAK state, was determined in the presence of 5 mM pyruvate, 2 mM malate, and 10 mM glutamate, but not adenylates [41,66]. The OXPHOS respiration was activated by the addition of saturating ADP concentration (2.5 mM), followed by supplementation with 10 mM succinate. The maximal ET capacity was observed by uncoupler titration using 0.5 μM of CCCP. The residual oxygen consumption, the ROX state, was finally achieved by inhibiting the ET chain with 2 μM rotenone and 2.5 μM antimycin.

Alternatively, HRR was used in intact cells to monitor changes in the ET capacity [67]. Briefly, after the stabilization of ROUTINE respiration, the maximal ET capacity was achieved by the addition of 1 μM of CCCP. Different concentrations of rotenone (1 to 16 nM) or malonic acid (0.1 to 1.6 µM) were directly added in the cuvette.

All experiments were performed in a Mir05 respiration buffer (Oroboros Instruments) at 37 °C under constant stirring. All chemicals were purchased from Sigma Aldrich (St. Louis, MO, USA).

### 4.6. Analysis of Respirometric States

Instrumental and chemical background fluxes were calibrated as a function of the oxygen concentration using DatLab software (v7.4.0.1, Oroboros Instruments). The rate of oxygen consumption corresponding to the ROUTINE, OXPHOS, and maximal ET capacity was corrected for the ROX and expressed as pmol/s per million cells. Flux control ratios (FCRs) relative to the maximal ET capacity, the reserve capacities, and the contribution of the N- and S-pathways to the respiratory profile were calculated as in [51,68].

### 4.7. Chromatographic Analysis of Energetic Metabolites

Analysis of coenzymes was driven on deproteinized samples following a method set up in our laboratories [69]. This protocol is eligible to protect labile compounds from oxidation or hydrolysis. Briefly, 1 mL of a precipitating solution (CH_3_CN + KH_2_PO_4_ 10 mM pH 7.4; 3:1 *v*:*v*) was added to 3 × 10^6^ pelleted cells, vigorously mixed, and centrifuged at a high speed (20,890× *g* for 10 min at 4 °C). The supernatant was supplemented with two volumes of chloroform to discharge acetonitrile and all hydrophobic contamination and centrifuged again under the conditions described above. Finally, an aqueous sample suitable for chromatographic separation was recovered. A P4000 pump (Thermo Electron, Waltham, MA, USA), drove HAP1 parental and ΔVDAC1 cellular extracts into a Hypersil column (250 × 4.6 mm, 5 µm particle size) according to a well-established binary gradient. Analyte identification and quantification were conducted using a highly sensitive UV6000LP diode array detector (Thermo Electron) equipped with a 5-cm light path flow cell and set up with a wavelength between 200 and 300 nm. A standard mixture run was used as reference control.

### 4.8. Determination of Intracellular Lactate

The intracellular lactate was tested by colorimetric assay as in [70]. Briefly, 50 µL of cellular extracts were mixed with a lactate oxidase/peroxidase-based reagent in Tris buffer and 4-aminoantipyrine to create a colored adduct. The absorbance variation, directly proportional to lactate concentration, followed in an automatic microplate photometer Elx800 Box 998 (BioTek Instruments, Winooski, VT, USA) set up at 545 nm wavelength. This variation was compared to a standard curve.

### 4.9. Statistical Analysis

All data are expressed as means or a median with standard deviation. A minimum of three independent experiments were performed for each assay. Data were statistically analyzed by *t*-test or two-way ANOVA using Prism software (GraphPad, San Diego, CA, USA). The following values * *p* < 0.05, ** *p* < 0.01, *** *p* < 0.001 were taken as significant.

## Data Availability

Not applicable.

## Supplementary Materials

The following supporting information can be downloaded at: https://www.mdpi.com/article/10.3390/ijms24043687/s1.

## References

[1] Shoshan-Barmatz V., De Pinto V., Zweckstetter M., Raviv Z., Keinan N., Arbel N. (2010). VDAC, a Multi-Functional Mitochondrial Protein Regulating Cell Life and Death. Mol. Asp. Med..

[2] De Pinto V. (2021). Renaissance of VDAC: New Insights on a Protein Family at the Interface between Mitochondria and Cytosol. Biomolecules.

[3] Forte M., Adelsberger-Mangan D., Colombini M. (1987). Purification and Characterization of the Voltage-Dependent Anion Channel from the Outer Mitochondrial Membrane of Yeast. J. Membr. Biol..

[4] Benz R., Wojtczak L., Bosch W., Brdiczka D. (1988). Inhibition of Adenine Nucleotide Transport through the Mitochondrial Porin by a Synthetic Polyanion. FEBS Lett..

[5] Nibali S.C., Di Rosa M.C., Rauh O., Thiel G., Reina S., De Pinto V. (2021). Cell-Free Electrophysiology of Human VDACs Incorporated into Nanodiscs: An Improved Method. Biophys. Rep..

[6] Messina A., Reina S., Guarino F., De Pinto V. (2012). VDAC Isoforms in Mammals. Biochim. Biophys. Acta Biomembr..

[7] Ujwal R., Cascio D., Colletier J.P., Faham S., Zhang J., Toro L., Ping P., Abramson J. (2008). The Crystal Structure of Mouse VDAC1 at 2.3 Å Resolution Reveals Mechanistic Insights into Metabolite Gating. Proc. Natl. Acad. Sci. USA.

[8] Hiller S., Garces R.G., Malia T.J., Orekhov V.Y., Colombini M., Wagner G. (2008). Solution Structure of the Integral Human Membrane Protein VDAC-1 in Detergent Micelles. Science.

[9] Bayrhuber M., Meins T., Habeck M., Becker S., Giller K., Villinger S., Vonrhein C., Griesinger C., Zweckstetter M., Zeth K. (2008). Structure of the Human Voltage-Dependent Anion Channel. Proc. Natl. Acad. Sci. USA.

[10] Schredelseker J., Paz A., López C.J., Altenbach C., Leung C.S., Drexler M.K., Chen J.N., Hubbell W.L., Abramson J. (2014). High Resolution Structure and Double Electron-Electron Resonance of the Zebrafish Voltage-Dependent Anion Channel 2 Reveal an Oligomeric Population. J. Biol. Chem..

[11] Manzo G., Serra I., Magrí A., Casu M., De Pinto V., Ceccarelli M., Scorciapino M.A. (2018). Folded Structure and Membrane Affinity of the N-Terminal Domain of the Three Human Isoforms of the Mitochondrial Voltage-Dependent Anion-Selective Channel. ACS Omega.

[12] De Pinto V., Guarino F., Guarnera A., Messina A., Reina S., Tomasello F.M., Palermo V., Mazzoni C. (2010). Characterization of Human VDAC Isoforms: A Peculiar Function for VDAC3?. Biochim. Biophys. Acta Bioenerg..

[13] Benz R. (1994). Permeation of Hydrophilic Solutes through Mitochondrial Outer Membranes: Review on Mitochondrial Porins. BBA—Rev. Biomembr..

[14] Rostovtseva T., Colombini M. (1997). VDAC Channels Mediate and Gate the Flow of ATP: Implications for the Regulation of Mitochondrial Function. Biophys. J..

[15] Gincel D., Shoshan-Barmatz V. (2004). Glutamate Interacts with VDAC and Modulates Opening of the Mitochondrial Permeability Transition Pore. J. Bioenerg. Biomembr..

[16] Camara A.K.S., Zhou Y.F., Wen P.C., Tajkhorshid E., Kwok W.M. (2017). Mitochondrial VDAC1: A Key Gatekeeper as Potential Therapeutic Target. Front. Physiol..

[17] Magrì A., Reina S., De Pinto V. (2018). VDAC1 as Pharmacological Target in Cancer and Neurodegeneration: Focus on Its Role in Apoptosis. Front. Chem..

[18] Nakashima R.A. (1989). Hexokinase-Binding Properties of the Mitochondrial VDAC Protein: Inhibition by DCCD and Location of Putative DCCD-Binding Sites. J. Bioenerg. Biomembr..

[19] Schindler A., Foley E. (2013). Hexokinase 1 Blocks Apoptotic Signals at the Mitochondria. Cell. Signal..

[20] Magrì A., Belfiore R., Reina S., Tomasello M.F., Di Rosa M.C., Guarino F., Leggio L., De Pinto V., Messina A. (2016). Hexokinase i N-Terminal Based Peptide Prevents the VDAC1-SOD1 G93A Interaction and Re-Establishes ALS Cell Viability. Sci. Rep..

[21] Shteinfer-Kuzmine A., Argueti S., Gupta R., Shvil N., Abu-Hamad S., Gropper Y., Hoeber J., Magrì A., Messina A., Kozlova E.N. (2019). A VDAC1-Derived N-Terminal Peptide Inhibits Mutant SOD1-VDAC1 Interactions and Toxicity in the SOD1 Model of ALS. Front. Cell. Neurosci..

[22] Magri A., Messina A. (2017). Interactions of VDAC with Proteins Involved in Neurodegenerative Aggregation: An Opportunity for Advancement on Therapeutic Molecules. Curr. Med. Chem..

[23] Reina S., De Pinto V. (2017). Anti-Cancer Compounds Targeted to VDAC: Potential and Perspectives. Curr. Med. Chem..

[24] Risiglione P., Zinghirino F., Di Rosa M.C., Magrì A., Messina A. (2021). Alpha-Synuclein and Mitochondrial Dysfunction in Parkinson’s Disease: The Emerging Role of VDAC. Biomolecules.

[25] Cheng E.H.Y., Sheiko T.V., Fisher J.K., Craigen W.J., Korsmeyer S.J. (2003). VDAC2 Inhibits BAK Activation and Mitochondrial Apoptosis. Science.

[26] Ma S.B., Nguyen T.N., Tan I., Ninnis R., Iyer S., Stroud D.A., Menard M., Kluck R.M., Ryan M.T., Dewson G. (2014). Bax Targets Mitochondria by Distinct Mechanisms before or during Apoptotic Cell Death: A Requirement for VDAC2 or Bak for Efficient Bax Apoptotic Function. Cell Death Differ..

[27] Chin H.S., Li M.X., Tan I.K.L., Ninnis R.L., Reljic B., Scicluna K., Dagley L.F., Sandow J.J., Kelly G.L., Samson A.L. (2018). VDAC2 Enables BAX to Mediate Apoptosis and Limit Tumor Development. Nat. Commun..

[28] Reina S., Checchetto V., Saletti R., Gupta A., Chaturvedi D., Guardiani C., Guarino F., Scorciapino M.A., Magrì A., Foti S. (2016). VDAC3 as a Sensor of Oxidative State of the Intermembrane Space of Mitochondria: The Putative Role of Cysteine Residue Modifications. Oncotarget.

[29] Queralt-Martín M., Bergdoll L., Teijido O., Munshi N., Jacobs D., Kuszak A.J., Protchenko O., Reina S., Magrì A., De Pinto V. (2020). A Lower Affinity to Cytosolic Proteins Reveals VDAC3 Isoform-Specific Role in Mitochondrial Biology. J. Gen. Physiol..

[30] Saletti R., Reina S., Pittalà M.G.G., Belfiore R., Cunsolo V., Messina A., De Pinto V., Foti S. (2017). High Resolution Mass Spectrometry Characterization of the Oxidation Pattern of Methionine and Cysteine Residues in Rat Liver Mitochondria Voltage-Dependent Anion Selective Channel 3 (VDAC3). Biochim. Biophys. Acta Biomembr..

[31] Reina S., Conti Nibali S., Tomasello M.F., Magrì A., Messina A., De Pinto V. (2022). Voltage Dependent Anion Channel 3 (VDAC3) Protects Mitochondria from Oxidative Stress. Redox Biol..

[32] Di Rosa M.C., Guarino F., Nibali S.C., Magrì A., De Pinto V. (2021). Voltage-Dependent Anion Selective Channel Isoforms in Yeast: Expression, Structure, and Functions. Front. Physiol..

[33] Magrì A., Di Rosa M.C., Orlandi I., Guarino F., Reina S., Guarnaccia M., Morello G., Spampinato A., Cavallaro S., Messina A. (2020). Deletion of Voltage-Dependent Anion Channel 1 Knocks Mitochondria down Triggering Metabolic Rewiring in Yeast. Cell. Mol. Life Sci..

[34] Brahimi-Horn M.C., Giuliano S., Saland E., Lacas-Gervais S., Sheiko T., Pelletier J., Bourget I., Bost F., Féral C., Boulter E. (2015). Knockout of Vdac1 Activates Hypoxia-Inducible Factor through Reactive Oxygen Species Generation and Induces Tumor Growth by Promoting Metabolic Reprogramming and Inflammation. Cancer Metab..

[35] Yang M., Sun J., Stowe D.F., Tajkhorshid E., Kwok W.M., Camara A.K.S. (2020). Knockout of VDAC1 in H9c2 Cells Promotes Oxidative Stress-Induced Cell Apoptosis through Decreased Mitochondrial Hexokinase II Binding and Enhanced Glycolytic Stress. Cell. Physiol. Biochem..

[36] Essletzbichler P., Konopka T., Santoro F., Chen D., Gapp B.V., Kralovics R., Brummelkamp T.R., Nijman S.M.B., Bürckstümmer T. (2014). Megabase-Scale Deletion Using CRISPR/Cas9 to Generate a Fully Haploid Human Cell Line. Genome Res..

[37] Pesta D., Gnaiger E. (2012). High-Resolution Respirometry: OXPHOS Protocols for Human Cells and Permeabilized Fibers from Small Biopsies of Human Muscle. Methods Mol. Biol..

[38] Ben-Hail D., Begas-Shvartz R., Shalev M., Shteinfer-Kuzmine A., Gruzman A., Reina S., De Pinto V., Shoshan-Barmatz V. (2016). Novel Compounds Targeting the Mitochondrial Protein VDAC1 Inhibit Apoptosis and Protect against Mitochondrial Dysfunction. J. Biol. Chem..

[39] Verma A., Pittala S., Alhozeel B., Shteinfer-Kuzmine A., Ohana E., Gupta R., Chung J.H., Shoshan-Barmatz V. (2022). The Role of the Mitochondrial Protein VDAC1 in Inflammatory Bowel Disease: A Potential Therapeutic Target. Mol. Ther..

[40] Evinova A., Cizmarova B., Hatokova Z., Racay P. (2020). High-Resolution Respirometry in Assessment of Mitochondrial Function in Neuroblastoma SH-SY5Y Intact Cells. J. Memb. Biol..

[41] Gnaiger E. (2009). Capacity of Oxidative Phosphorylation in Human Skeletal Muscle. New Perspectives of Mitochondrial Physiology. Int. J. Biochem. Cell Biol..

[42] Yang Y., Sauve A.A. (2016). NAD^+^ Metabolism: Bioenergetics, Signaling and Manipulation for Therapy. Biochim. Biophys. Acta Proteins Proteom..

[43] Sherer T.B., Betarbet R., Testa C.M., Seo B.B., Richardson J.R., Kim J.H., Miller G.W., Yagi T., Matsuno-Yagi A., Greenamyre J.T. (2003). Mechanism of Toxicity in Rotenone Models of Parkinson’s Disease. J. Neurosci..

[44] Gonçalves R.P., Buzhynskyy N., Prima V., Sturgis J.N., Scheuring S. (2007). Supramolecular Assembly of VDAC in Native Mitochondrial Outer Membranes. J. Mol. Biol..

[45] Gonçalves R.P., Buzhysnskyy N., Scheuring S. (2008). Mini Review on the Structure and Supramolecular Assembly of VDAC. J. Bioenerg. Biomembr..

[46] Morgenstern M., Stiller S.B., Lübbert P., Peikert C.D., Dannenmaier S., Drepper F., Weill U., Höß P., Feuerstein R., Gebert M. (2017). Definition of a High-Confidence Mitochondrial Proteome at Quantitative Scale. Cell Rep..

[47] Seitaj B., Maull F., Zhang L., Wüllner V., Wolf C., Schippers P., la Rovere R., Distler U., Tenzer S., Parys J.B. (2020). Transmembrane BAX Inhibitor-1 Motif Containing Protein 5 (TMBIM5) Sustains Mitochondrial Structure, Shape, and Function by Impacting the Mitochondrial Protein Synthesis Machinery. Cells.

[48] Jastroch M., Divakaruni A.S., Mookerjee S., Treberg J.R., Brand M.D. (2010). Mitochondrial Proton and Electron Leaks. Essays Biochem..

[49] Porter R.K. (2001). Mitochondrial Proton Leak: A Role for Uncoupling Proteins 2 and 3?. Biochim. Biophys. Acta Bioenerg..

[50] Cannon B., Shabalina I.G., Kramarova T.V., Petrovic N., Nedergaard J. (2006). Uncoupling Proteins: A Role in Protection against Reactive Oxygen Species-or Not?. Biochim. Biophys. Acta Bioenerg..

[51] Risiglione P., Leggio L., Cubisino S.A.M., Reina S., Paternò G., Marchetti B., Magrì A., Iraci N., Messina A. (2020). High-Resolution Respirometry Reveals Mpp+ Mitochondrial Toxicity Mechanism in a Cellular Model of Parkinson’s Disease. Int. J. Mol. Sci..

[52] Calabria E., Scambi I., Bonafede R., Schiaffino L., Peroni D., Potrich V., Capelli C., Schena F., Mariotti R. (2019). Ascs-Exosomes Recover Coupling Efficiency and Mitochondrial Membrane Potential in an in Vitro Model of Als. Front. Neurosci..

[53] Magrì A., Risiglione P., Caccamo A., Formicola B., Tomasello M.F., Arrigoni C., Zimbone S., Guarino F., Re F., Messina A. (2021). Small Hexokinase 1 Peptide against Toxic SOD1 G93A Mitochondrial Accumulation in ALS Rescues the ATP-Related Respiration. Biomedicines.

[54] Cheng J., Nanayakkara G., Shao Y., Cueto R., Wang L., Yang W.Y., Tian Y., Wang H., Yang X. (2017). Mitochondrial Proton Leak Plays a Critical Role in Pathogenesis of Cardiovascular Diseases. Adv. Exp. Med. Biol..

[55] Lemieux H., Semsroth S., Antretter H., Höfer D., Gnaiger E. (2011). Mitochondrial Respiratory Control and Early Defects of Oxidative Phosphorylation in the Failing Human Heart. Int. J. Biochem. Cell Biol..

[56] Risiglione P., Cubisino S.A.M., Lipari C.L.R., De Pinto V., Messina A., Magrì A. (2022). α-Synuclein A53T Promotes Mitochondrial Proton Gradient Dissipation and Depletion of the Organelle Respiratory Reserve in a Neuroblastoma Cell Line. Life.

[57] Guardiani C., Magrì A., Karachitos A., di Rosa M.C., Reina S., Bodrenko I., Messina A., Kmita H., Ceccarelli M., de Pinto V. (2018). YVDAC2, the Second Mitochondrial Porin Isoform of Saccharomyces Cerevisiae. Biochim. Biophys. Acta Bioenerg..

[58] Magrì A., Karachitos A., Di Rosa M.C., Reina S., Conti Nibali S., Messina A., Kmita H., De Pinto V. (2019). Recombinant Yeast VDAC2: A Comparison of Electrophysiological Features with the Native Form. FEBS Open Bio.

[59] Magrì A., Di Rosa M.C., Tomasello M.F., Guarino F., Reina S., Messina A., De Pinto V. (2016). Overexpression of Human SOD1 in VDAC1-Less Yeast Restores Mitochondrial Functionality Modulating Beta-Barrel Outer Membrane Protein Genes. Biochim. Biophys. Acta Bioenerg..

[60] Zinghirino F., Pappalardo X.G., Messina A., Nicosia G., De Pinto V., Guarino F. (2021). VDAC Genes Expression and Regulation in Mammals. Front. Physiol..

[61] Cesar M.D.C., Wilson J.E. (2004). All Three Isoforms of the Voltage-Dependent Anion Channel (VDAC1, VDAC2, and VDAC3) Are Present in Mitochondria from Bovine, Rabbit, and Rat Brain. Arch. Biochem. Biophys..

[62] Leggio L., Guarino F., Magrì A., Accardi-Gheit R., Reina S., Specchia V., Damiano F., Tomasello M.F., Tommasino M., Messina A. (2018). Mechanism of Translation Control of the Alternative Drosophila Melanogaster Voltage Dependent Anion-Selective Channel 1 MRNAs. Sci. Rep..

[63] Reina S., Palermo V., Guarnera A., Guarino F., Messina A., Mazzoni C., De Pinto V. (2010). Swapping of the N-Terminus of VDAC1 with VDAC3 Restores Full Activity of the Channel and Confers Anti-Aging Features to the Cell. FEBS Lett..

[64] Pronevich L.A., Mirzabekov T.A., Rozhdestvenskaya Z.E. (1989). Mitochondrial porin regulates the sensitivity of anion carriers to inhibitors. FEBS Lett..

[65] Leggio L., L’episcopo F., Magrì A., Ulloa-Navas J., Paternò G., Vivarelli S., Bastos C.A.P., Tirolo C., Testa N., Caniglia S. (2022). Small Extracellular Vesicles Secreted by Nigrostriatal Astrocytes Rescue Cell Death and Preserve Mitochondrial Function in Parkinson’s Disease. Adv. Healthc. Mater..

[66] Chance B., Williams G.R. (1955). Respiratory Enzymes in Oxidative Phosphorylation. III. The Steady State. J. Biol. Chem..

[67] Bețiu A.M., Chamkha I., Gustafsson E., Meijer E., Avram V.F., Frostner E.Å., Ehinger J.K., Petrescu L., Muntean D.M., Elmér E. (2021). Cell-permeable Succinate Rescues Mitochondrial Respiration in Cellular Models of Amiodarone Toxicity. Int. J. Mol. Sci..

[68] Gnaiger E. (2020). Mitochondrial pathways and respiratory control. An introduction to OXPHOS analysis. Bioenerg. Commun..

[69] Lazzarino G., Amorini A.M., Fazzina G., Vagnozzi R., Signoretti S., Donzelli S., di Stasio E., Giardina B., Tavazzi B. (2003). Single-Sample Preparation for Simultaneous Cellular Redox and Energy State Determination. Anal. Biochem..

[70] Artiss J.D., Karcher R.E., Cavanagh K.T., Collins S.L., Peterson V.J., Varma S., Zak B. (2000). A Liquid-Stable Reagent for Lactic Acid Levels: Application to the Hitachi 911 and Beckman CX7. Am. J. Clin. Pathol..

