# Supplementary material for: VDAC1 Knockout Affects Mitochondrial Oxygen Consumption Triggering a Rearrangement of ETC by Impacting on Complex I Activity
Source: Int J Mol Sci. 2023 Feb 12;24(4):3687. doi: 10.3390/ijms24043687 (PMC9963415; doi:10.3390/ijms24043687)
Supplement: Supplementary file 1 [file ijms-24-03687-s001.zip › ijms-2047857-supplementary.pdf]

Supplementary Materials

VDAC1 Knockout Affects Mitochondrial Oxygen Consumption Triggering a Rearrangement of ETC by Impacting on Complex I Activity

Andrea Magrì[1,2,†,*], Salvatore Antonio Maria Cubisino[3,†], Giuseppe Battiato[3], Cristiana Lucia Rita Lipari[3], Stefano Conti Nibali[3], Miriam Wissam Saab[4], Alessandra Pittalà[4], Angela Maria Amorini[4], Vito De Pinto[2,3], Angela Messina[1,2]

[1] Department of Biological, Geological and Environmental Sciences, University of Catania, Via S. Sofia 64, 95125 Catania, Italy
[2] we.MitoBiotech S.R.L., C.so Italia 174, 95125 Catania, Italy.
[3] Department of Biomedical and Biotechnological Sciences, University of Catania, Via S. Sofia 64, 95125 Catania, Italy
[4] Department of Biomedical and Biotechnological Sciences, Division of Medical Biochemistry, University of Catania, Via S.Sofia 97, 95123 Catania, Italy
* Correspondence: andrea.magri@unict.it.
† These authors have equally contributed

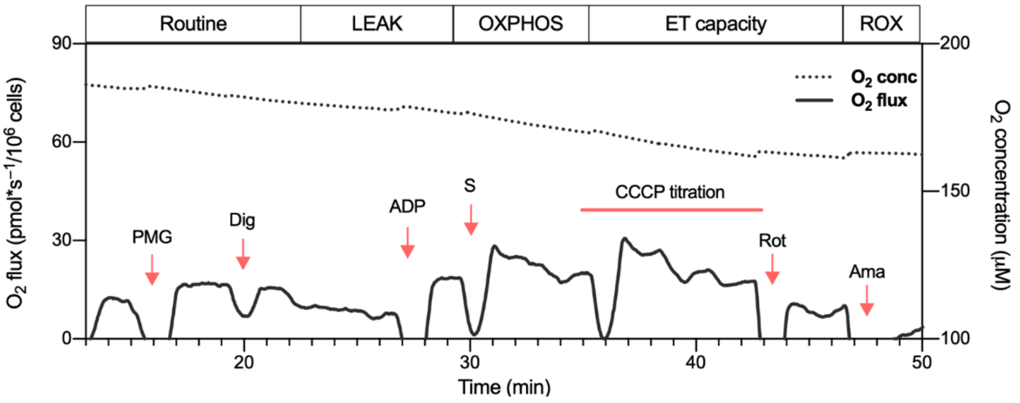

Figure S1. Oxygen consumption in HAP1 VDAC1 knock-out cells. A representative curve of mitochondrial respiratory profile of HAP1 ΔVDAC1 cells along with the SUIT protocol used in this work. P, pyruvate; M, malate; G, glutamate; Dig, digitonin; S, succinate; Rot, rotenone; Ama, antimycin.

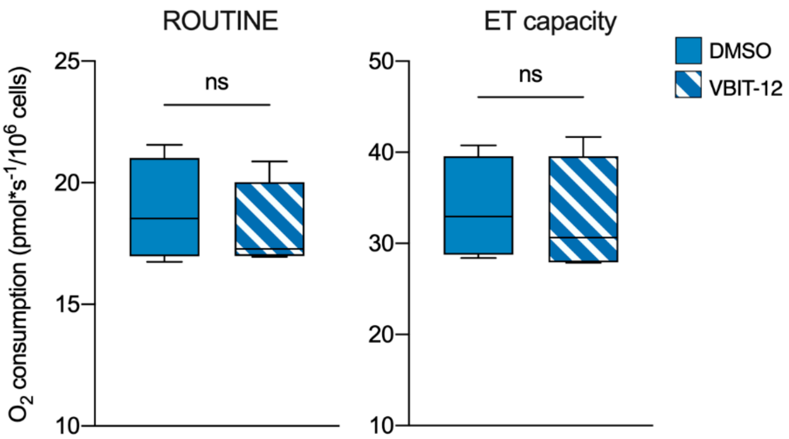

Figure S2. Analysis of VBIT-12 effect on respiration of ΔVDAC1 cells. Quantitative analysis of the oxygen consumption rates of ROUTINE and maximal ET capacity in HAP1 ΔVDAC1 cells previously treated with VBIT-12 or DMSO (control). No significant variation has been observed for both ROUTINE or ET capacity. Data are expressed as pmol/second per million cells and shown as median ± SEM of n=6 independent experiments.

Table S1. Raw data of the nicotinamide dinucleotides expressed as nmol/million cells. Data are relative to the quantification of each nicotinamide dinucleotide in HAP1 parental and VDAC1 knockout cells. Data are expressed as means ± SD of n=4 independent measurements.

	**NAD^+**	**NADH**	**$NADP^+$**	**NADPH**
Parental	1.48 ± 0.64	0.18 ± 0.04	0.42 ± 0.19	0.11 ± 0.04
ΔVDAC1	0.87 ± 0.44	0.09 ± 0.41	0.22 ± 0.06	0.14 ± 0.04
